# How did I miss that? Developing mixed hybrid visual search as a ‘model system’ for incidental finding errors in radiology

**DOI:** 10.1186/s41235-017-0072-5

**Published:** 2017-08-23

**Authors:** Jeremy M. Wolfe, Abla Alaoui Soce, Hayden M. Schill

**Affiliations:** 1 Ophthalmology and Radiology Departments, Harvard Medical School, 64 Sidney St. Suite 170, Cambridge, MA 02139 USA; 2 Visual Attention Lab, Brigham and Women’s Hospital, 64 Sidney St. Suite 170, Cambridge, MA 02139 USA

**Keywords:** Visual search, Hybrid search, Radiology, Incidental findings, Errors

## Abstract

In a real world search, it can be important to keep ‘an eye out’ for items of interest that are not the primary subject of the search. For instance, you might look for the exit sign on the freeway, but you should also respond to the armadillo crossing the road. In medicine, these items are known as “incidental findings,” findings of possible clinical significance that were not the main object of search. These errors (e.g., missing a broken rib while looking for pneumonia) have medical consequences for the patient and potential legal consequences for the physician. Here we report three experiments intended to develop a ‘model system’ for incidental findings – a paradigm that could be used in the lab to develop strategies to reduce incidental finding errors in the clinic. All the experiments involve ‘hybrid’ visual search for any of several targets held in memory. In this ‘mixed hybrid search task,’ observers search for any of three specific targets (e.g., this rabbit, this truck, and this spoon) and three categorical targets (e.g., masks, furniture, and plants). The hypothesis is that the specific items are like the specific goals of a real world search and the categorical targets are like the less well-defined incidental findings that might be present and that should be reported. In all these experiments, varying target prevalence, number of targets, etc., the categorical targets are missed at a much higher rate than the specific targets. This paradigm shows promise as a model of the incidental finding problem.

## Significance

Incidental findings are a significant issue in clinical radiology. They are difficult to study in a systematic fashion because both the stimulus material and the observer population are hard to assemble. Here we propose a “model system”, in the form of a ‘mixed hybrid search’ task. It can be used to investigate the fundamental cognitive processes that lie behind incidental finding errors. Once those principles are identified, tractable experiments can be designed for clinical settings with expert observers.

## Background

A radiologist is asked to read a chest X-ray to determine if a patient has pneumonia. After assessing the exam, he decides that she does not. He is correct; she does not have pneumonia, but she does have clear signs of lung cancer that the radiologist fails to report. The radiologist has missed an “incidental finding” (Beigelman-Aubry, Hill, & Grenier, 2007).

Incidental findings are items of potential clinical significance that may not have been the primary object of the search of the image. In one review, incidental findings appeared on 24% of a mixed collection of radiologic cases (Lumbreras, Donat, & Hernández-Aguado, 2010). Not all such findings turn out to be important. The vigor with which incidental findings should be reported and followed up is debatable (Berlin, 2016; Pandharipande et al., 2016a, 2016b). A recent study of head computed tomography (CT) from 5800 patients described possible incidental findings in about 10% of cases, followed up on about 3% and found that most of those were “without direct clinical consequences” (Bos et al., 2016). Nevertheless, there are cases where the missed finding is clinically significant and where failure to report the finding can have adverse consequences for the patient as well as for the clinician, in the form of a malpractice suit. Radiologists know that the search for these incidental targets is part of the task and should be considered whenever they look at an image.

Very similar problems occur outside of the medical field as well. If you are about to cross the street and you look both ways for cars only to be very nearly knocked down by a bicycle, you have, arguably, committed the same type of error. You knew that your task was to look for anything that might have direct consequence on your ability to safely cross the street, yet you failed to respond to the bicycle (which, for purposes of argument, we will assume was clearly visible). How can we better understand the processes behind this common problem and how should we try to ameliorate it? This class of real-world problems is difficult to study in the real world. If we stay with the radiology example, it is possible to retrospectively study the issue; for example, by doing a second reading of a set of cases, specifically looking for incidental findings. However, it is neither practical nor ethical to manipulate variables in the clinic simply on the hunch that they might alter the rate of incidental findings found. Rather, like other problems in medicine, we need a model system that can be studied extensively in the lab before proposing more limited, evidence-based hypotheses that can be tested in the clinic (or the bike lane). The purpose of this paper is to propose one such model system, “mixed hybrid search,” in which observers search a visual display for some specific targets (e.g., “this rabbit”) and some general targets (e.g., “any vehicle”). As we will describe, the chance of missing a general target can be markedly elevated in mixed hybrid tasks, perhaps in a manner similar to the elevated rate with which incidental findings are missed.

Before describing the mixed hybrid model system, it is worth discussing two other possible models that have been extensively studied in recent years: inattentional blindness (Mack & Rock, 1998) and satisfaction of search (Berbaum et al., 1990; Tuddenham, 1962). Perhaps the most famous example of inattentional blindness is the Simons and Chabris (1999) gorilla experiment. In that experiment, observers are asked to count the number of times the white-shirted team touches the ball in a ball-passing game. About half of those observers fail to report an actor in a gorilla suit walk through the middle of the game. The phenomenon has been extensively researched (Cohen, Cavanagh, Chun, & Nakayama, 2012) with extensions to other senses (audition: Dalton & Fraenkel, 2012) and to various real-world settings (Castel, Vendetti, & Holyoak, 2012; Chabris & Simons, 2011). Our lab explicitly connected the phenomenon to the incidental finding problem by placing an image of a gorilla in a lung CT and showing that expertise did not immunize radiologists against inattentional blindness. Twenty of 24 radiologists failed to report the gorilla (Drew, Vo, & Wolfe, 2013).

The difficulty with inattentional blindness as a model of incidental findings is that no radiologist is looking for a gorilla in the lung, even incidentally. Nor were Simons and Chabris (1999) observers looking for a gorilla. Indeed, the effect goes away if observers are told to count ball passes and to keep an eye open for the occasional gorilla. Incidental findings, in contrast, are targets that plausibly could be present and that should be kept in mind, but are often missed, nevertheless.

The phenomenon of satisfaction of search involves missing targets that the observer is, in fact, looking for. The problem was originally described in radiology when it was discovered that finding one target (e.g., a fracture) made it less likely that a second target in the same image would be found (Berbaum et al., 2001). The problem was dubbed “satisfaction of search” by Tuddenham (1962), based on the hypothesis that observers were “satisfied” by finding the first target and abandoned the search too quickly thereafter. Berbaum et al. (1991) subsequently showed that the rapid quitting idea was not correct. Nevertheless, the term persists though Adamo, Cain, and Mitroff (2013) have proposed “Subsequent Search Misses (SSM)” as a theory-neutral term. A significant body of work exists in both medical image perception (reviewed in Berbaum, Franken, Caldwell, & Shartz, 2010) and in the basic visual cognition literature (Cain, Adamo, & Mitroff, 2013). Nevertheless, like inattentional blindness, satisfaction of search is not quite the right model system for incidental findings. There are two problems. First, the missed, second target, is typically of the same type as the first target: two fractures, two lung nodules, etc. Incidental findings are typically of a different type than the primary target of search: look for pneumonia, miss the cancer. Second, by definition, the error in satisfaction of search is the missing of a second target in an image. An incidental finding can be the only clinically significant finding in the image, but missed nevertheless.

Our goal is to create a model system for studying incidental findings in which observers know what they are looking for but, nevertheless, show elevated error rates for one class of stimuli that serve as our stand-in for incidental findings. To do this, we had observers search for a mixture of specific and categorical target types. The logic of this mixture approach is that the observer will know the nature of the targets (no surprise gorillas). We know that attention can be guided to categorical targets (Nako, Wu, Smith, & Eimer, 2014; Yang & Zelinsky, 2009), but we would expect observers to be less precise in their search for these less precise, categorical target (Maxfield & Zelinsky, 2012). We would expect them to miss more categorical than specific items. This elevated error rate can, then, stand in for the incidental finding errors we are trying to model.

Search tasks that have observers searching for multiple types of targets in a visual search display are known as “hybrid search” tasks (Schneider & Shiffrin, 1977; Wolfe, 2012a): “Hybrid” because they combine visual search with memory search in the same task. Using photographic images of specific objects, Wolfe (2012a, 2012b) found that hybrid search was characterized by reaction times (RTs) that were a linear function of the visual set size – the number of items in the visual display. The RTs were a linear function of the log of the memory set size – the number of target types held in memory. In these experiments, observers typically learn a memory set of target items and then search for members of that set in a block of several hundred trials. Different patterns of results are found if observers learn new targets on each trial (Nosofsky, Cao, Cox, & Shiffrin, 2014; Nosofsky, Cox, Cao, & Shiffrin, 2013). The logarithmic relationship between memory set size and hybrid search RT holds for large memory set sizes of 100 (Wolfe, 2012a) or even 500 specific items (Wolfe, Boettcher, Josephs, Cunningham, & Drew, 2015) and appears to be based on the recognition of items as targets rather than a more basic feeling of ‘familiarity’ (Wolfe, Boettcher, Josephs, Cunningham, & Drew, 2015). A similar pattern of results is seen with other types of targets such as words (Boettcher & Wolfe, 2015).

**Fig. 1:**
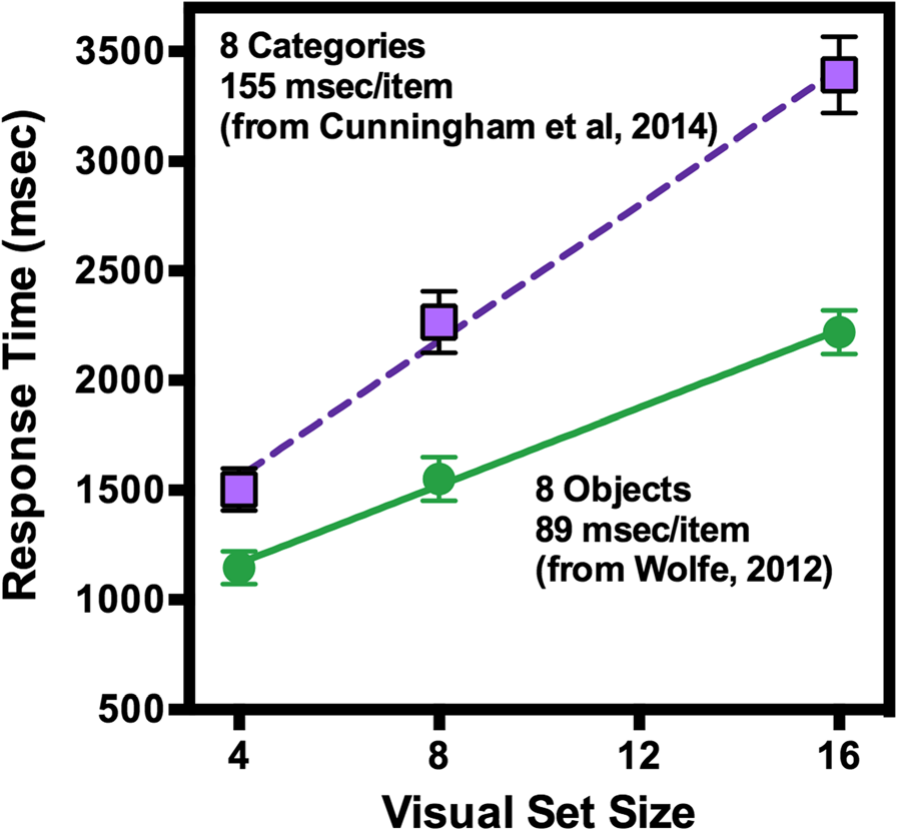
Hybrid search for eight specific or categorical target types. Data are redrawn from Wolfe (2012a, 2012b) and Cunningham and Wolfe (2014)

Importantly for present purposes, the same pattern of results is seen when broad categories are used as stimuli (Cunningham & Wolfe, 2014). Observers cannot easily memorize 100 categories in the way that they can memorize 100 specific objects. However, in Cunningham and Wolfe (2014), observers could easily memorize 1–8 categories like “plants, furniture, animals, weapons, picture frames, signs, flags, and cars.” Results again showed a linear relationship of RT to the visual set size and to the log of the memory set size. Searching for categorical targets is markedly more difficult that searching for specific targets. This is illustrated in Fig. 1.

The figure shows average RTs for target-present trials with a memory set of eight target types and visual set sizes of four, eight, and 16 items. These are extracted from larger data sets for illustrative purposes from Wolfe (2012a, 2012b) in the case of specific target types and Cunningham and Wolfe (2014) in the case of categorical target types. Error rates are low (<10%) in both conditions.

Clearly, the categorical task is slower than the specific task. Suppose that we mixed target types. That is, on any given trial, the target could be one of four specific objects or a member of one of four categories. It could be that the internal process of testing if the current visual item belongs to this memory set of eight items becomes as slow as the search for eight categorical target types. It could be that, as in Fig. 1, the specific targets are identified quickly and the categorical targets are found slowly. If that is the case, what happens when no target is found? Search termination seems to involve setting an internal quitting threshold based on experience with finding targets (Chun & Wolfe, 1996; Moran, Zehetleitner, Liesefeld, Müller, & Usher, 2015; Schwarz & Miller, 2016; Wolfe, 2012a, 2012b). With two different types of targets, the quitting threshold could reflect the time to find the harder targets. However, if the quitting time was substantially influenced by a contribution from the easier targets, observers might quit relatively quickly and, as a consequence, they might miss a relatively high proportion of the more difficult, categorical targets. This is, in fact, what the data show.

## Experiment 1: Mixed specific and categorical targets

### Participants

Twelve observers were tested (11 females, average age 23). Our main interest here is the error rates. Taking a typical experiment from Cunningham and Wolfe (2014), we have 4% errors with a 2% standard deviation. With six observers, we would be able to detect a doubling of the error to 8% with a power of 0.8 and a Type 1 error of 1%. We tested 12 observers. All observers gave informed consent and were paid at a rate of $10/hour. For all experiments in this paper, informed consent procedures were approved by the Partners Human Research Committee, protocol 2007P000646/BWH. All observers had at least 20/25 vision with correction, all passed the Ishihara Color Test, and all were fluent speakers and readers of English.

### Methods

In this experiment, observers searched for specific items, categorical items, or both, in the critical, mixed condition. There were six blocks of trials, differentiated by the memory set of possible target items. Observers could be asked to look for any of three specific target types, three categorical target types, six specific, six categorical, or a mixture of three specific and three categorical target types. This critical, mixed condition was repeated twice. The order of blocks was counterbalanced across subjects. All stimuli were photographs of isolated objects (provided by Talia Konkle). Available categories were: Animals, Cars, Hats, Masks, Shoes, Fruit, Furniture, Kitchenware, Musical Instruments, Plans, Rocks and Minerals, Signs, Sweets, Time Pieces, and Weapons. For each of the fifteen categories, we had 50 images.

At the start of each block, observers learned the memory set by viewing each item and/or the name of the category for 3 seconds. They were then presented with a series of images to test their knowledge of the memory set and were allowed to proceed to the hybrid visual search portion of the task only when they had correctly categorized 100% of the items as targets or distractors. After passing the memory test, observers performed 30 practice and 300 experimental visual search trials. For the mixed case, with a memory set of three categorical and three specific target types, observers were tested for 600 experimental trials in two separate blocks of 300 trials so as to provide 300 specific and 300 categorical trials in that condition. Trials were evenly divided between target-present and target-absent trials. Visual set sizes (items on the screen) were evenly divided between four, eight and 16 items. Observers were asked to be as fast and accurate as possible. Feedback about accuracy was given on a trial by trial basis.

### Results

**Fig. 2:**
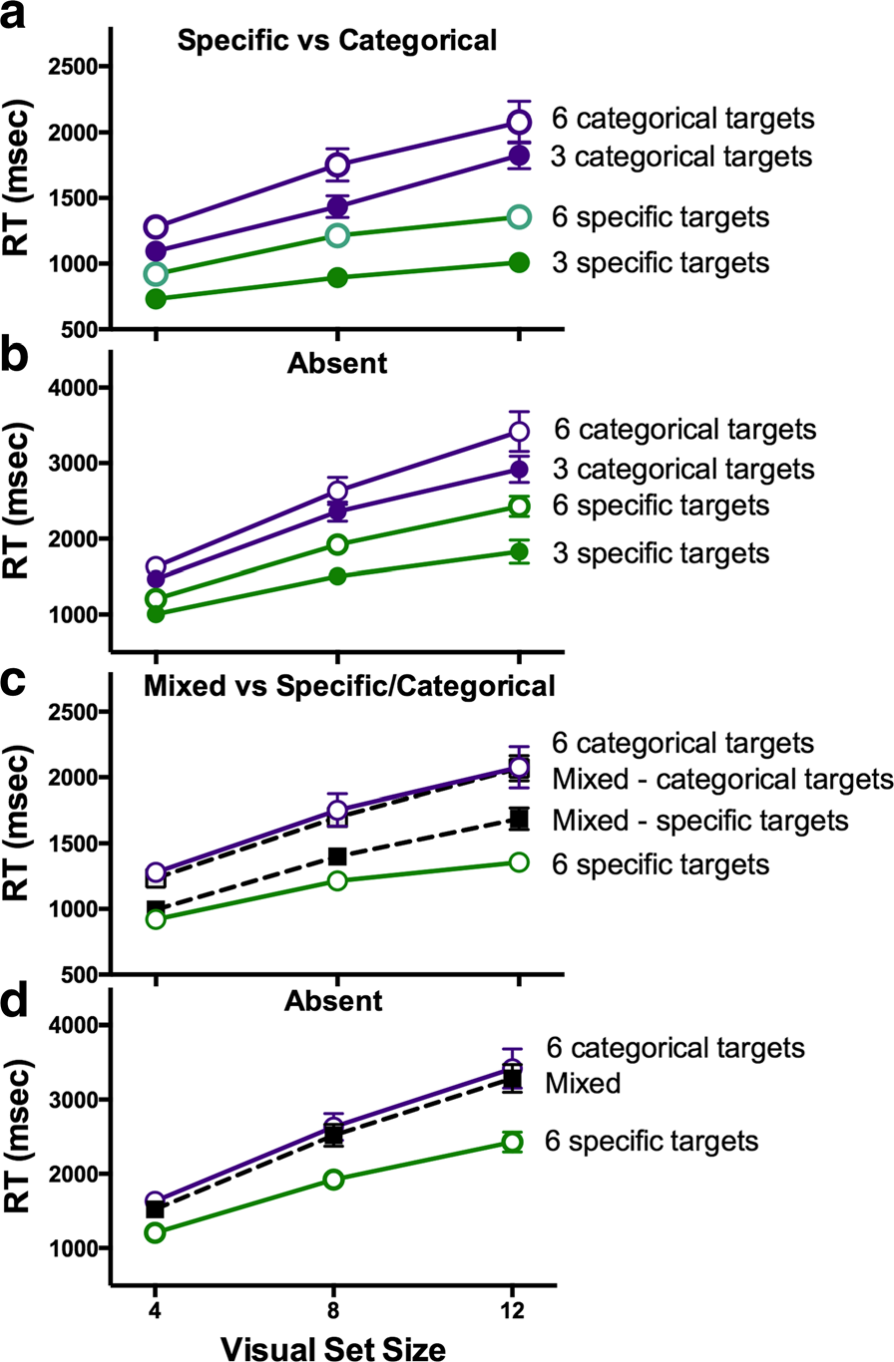
Reaction time (RT) as a function of visual set size in Experiment 1. Of most importance for present purposes, note that search for categorical targets is slower and less efficient than search for specific targets. Panels **a** and **b** show the RT data for the categorical vs. specific target conditions in present and absent trials (respectively). Panels **c** and **d** show RT data for the categorical and specific targets the Mixed condition (black) for present and absent trials (respectively). Data for 6 categorical, 6 specific conditions are replotted for comparison. Error bars represent +/- 1 standard error of the mean (s.e.m.)

Average RTs are plotted in Fig. 2. RTs were removed from analysis if they were less than 200 msec or greater than 10,000 msec. This removed 0.6% of the trials, the majority of which were due to a programming error that removed 84 trials from one observer’s data. Two sets of comparisons are shown in Fig. 2. Figure 2a and b show the standard hybrid search results for memory sets of three and six specific or categorical targets. Present trials are shown in 2a; absent in 2b.

These results replicate prior findings (Cunningham & Wolfe, 2014; Wolfe, 2012a, 2012b). Observers respond faster to targets from a memory set of three than from a memory set of six, and they are faster to find specific targets than categorical targets. Figure 2c and d replot the results for memory set size of six and add the results for the mixed condition that had three specific and three categorical targets. RTs are plotted separately for trials where the target was specific and those where it was categorical. Figure 2c shows that, on target present trials, the specific targets in the mixed condition are somewhat slower than search for any of six specific targets. The categorical targets in the mixed condition take about the same amount of time to find as search for any of six categorical targets. There is only one kind of mixed absent trial. As shown in Fig. 2d, these RTs are slower than the absent trials in the specific block and slightly faster than the absent trials in the categorical block.

If we treat the specific and categorical trials in the mixed blocks as separate conditions, there are six conditions: Memory set size three: Specific and Categorical, Memory set size six: Specific and Categorical, and Mixed: Specific and Categorical, Taking all six conditions together, an ANOVA reveals strong main effects of the condition (F(5,55) = 36.6, *p* < 0.0001_,_ generalized eta-squared (ges) = 0.56), a strong effect of visual set size (F(2,22) = 205.9, *p* < 0.0001,_,_ ges = 0.51), and a strong interaction (F(10,110) = 9.3, *p* < 0.0001,_,_ ges = 0.10). The pattern is essentially the same for absent trials and correction for violations of sphericity changes nothing substantial.

**Fig. 3:**
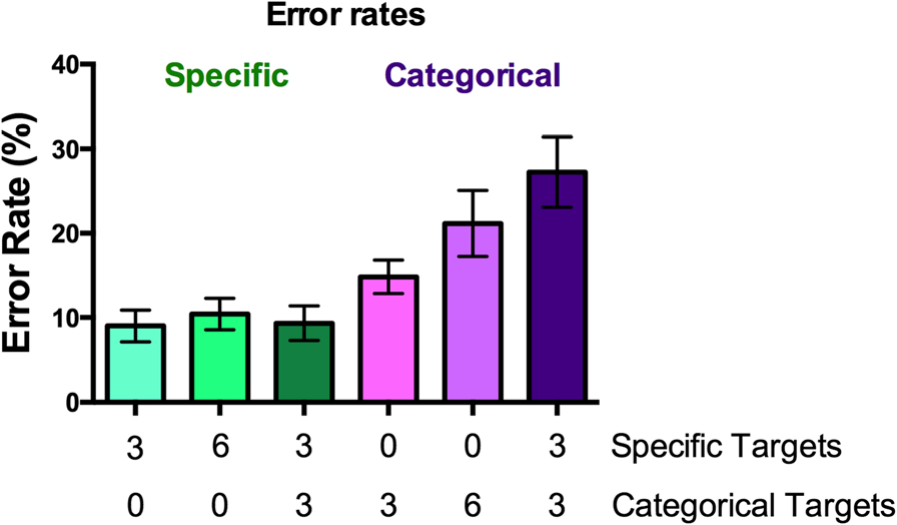
Miss error rates for Experiment 1. Note that error rates are higher for categorical targets, especially in the case where categorical and specific targets appear in the same block (columns 3 and 6). Error bars represent +/- 1 s.e.m

In this experiment, our primary interest lies in the miss error rates. These are shown in Fig. 3 for the six conditions. Specifically, the incidental finding prediction is that the categorical targets in the mixed condition would be missed at the highest rate. Figure 3 shows that this is indeed the case.

Statistical analysis of errors was performed on arcsine transformed error rates (Hogg & Craig, 1995). *T* tests are corrected for multiple comparisons, yielding a corrected alpha of 0.0033 if we were comparing all six error rates to each other at a *p* = 0.05 level. Note that this is conservative because, in fact, not all of the paired comparisons are of interest. Nevertheless, under these conditions, the categorical targets in the mixed condition are missed significantly more often than any of the other conditions except for the six categorical target condition. For this comparison, t(11) = 3.27, *p* = 0.0037; it is very close to, but not quite achieving the conservative *p* = 0.0033 level. The most informative comparison in the error data is the comparison between specific and categorical targets in the mixed conditions (columns 3 and 6 of Fig. 3). Specific targets were missed 9% of the time while categorical targets in the same block of trials were missed 27% of the time (t(11) = 5.68, *p* = 0.0001). Thus, the mixed hybrid search condition mimics the incidental finding problem. Members of a class of targets, in this case, categorical targets, are missed more often even though observers are looking for those targets.

## Experiment 2: Varying the relative prevalence of specific and categorical targets

In the real incidental finding situation, the items that are likely to be missed are not only a different type of target, they also appear less frequently in the population. Thus, if you are checking images of the lung for pneumonia, you should report lung cancer if it is present, but, not only is it not your specific target, it is also probably less likely to appear in a collection of patients suspected of having pneumonia. In Experiment 2, we repeat the mixed hybrid search experiment with a systematic variation in the prevalence of the specific and categorical targets.

### Methods

All of the conditions in Experiment 2 were mixed conditions with three specific and three categorical targets. The relative proportion of each type of target varied across three conditions. In the 20/80 condition, 20% of targets were categorical and 80% were specific. Since targets were present on 50% of trials, this means that overall, 10% of trials had categorical targets, and 40% of trials had specific targets. The other two conditions were 50/50 and 80/20. Visual set sizes were four, eight, and 12. Observers were tested for 60 practice and 600 experimental trials, evenly divided between present and absent trials and evenly divided over the three set sizes. In all other respects, the experiment was similar to Experiment 1. Twelve observers completed the experiment (eight females, ages 21–52). All gave informed consent and were paid $10/hour.

### Results and Discussion

Trials with RTs < 200 msec or greater than 10,000 msec were eliminated. This removed just 0.4% of trials.

**Fig. 4:**
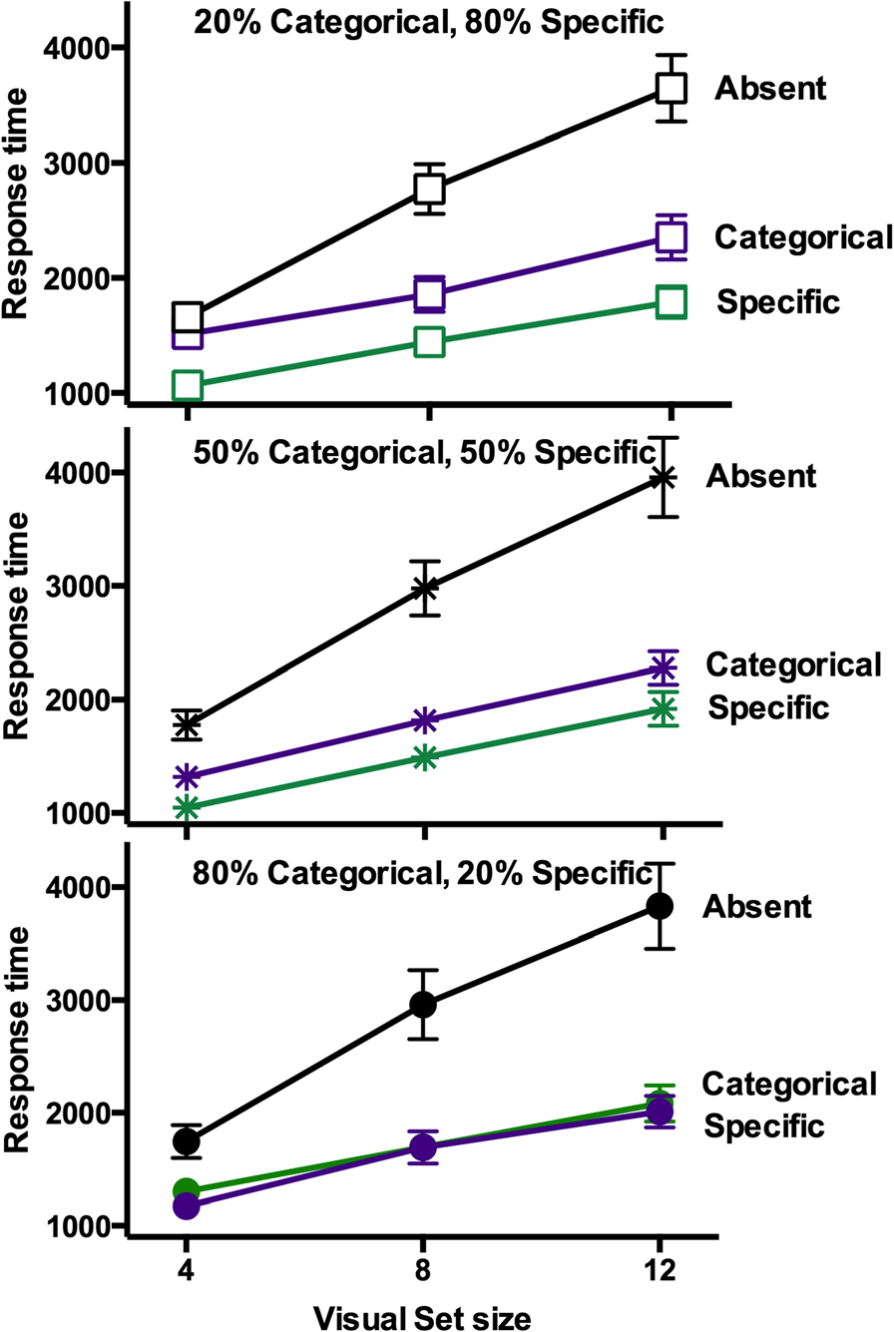
RT as a function of visual set size in Experiment 2. Note again that search for categorical targets is slower and less efficient than search for specific targets, except when categorical targets are much more common than specific targets. Error bars represent +/- 1 s.e.m

Figure 4 shows the RT × set size functions for each of the conditions with RTs for specific and categorical targets shown separately. An ANOVA with condition (specific target, categorical target, and absent) and visual set size as factors shows large effects of condition (F(4,44) = 30.8, *p* < 0.0001_,_ ges = 0.42), a strong effect of visual set size (F(2,22) = 177.0, *p* < 0.0001_,_ ges = 0.60), and a strong interaction (F(8,88) = 15.5, *p* < 0.0001_,_ ges = 0.09).

Again, it is the miss errors that are of prime interest and the critical condition is the condition in which the categorical targets are rare: the 20/80 condition. In that condition, observers missed just 5% of the specific targets and 37.5% of the categorical targets (t(11) = 20.6, *p* < 0.0001). The 50/50 case replicates the equivalent condition of the previous experiment: Specific errors constituted 9% of errors, categorical errors 23% (t(11) = 7.5, *p* < 0.0001). Only when categorical targets are four times more common than specific targets does the difference go away: Categorical errors drop to 14% compared to 17% specific errors (11) = 1.4, *p* < 0.18).

**Fig. 5:**
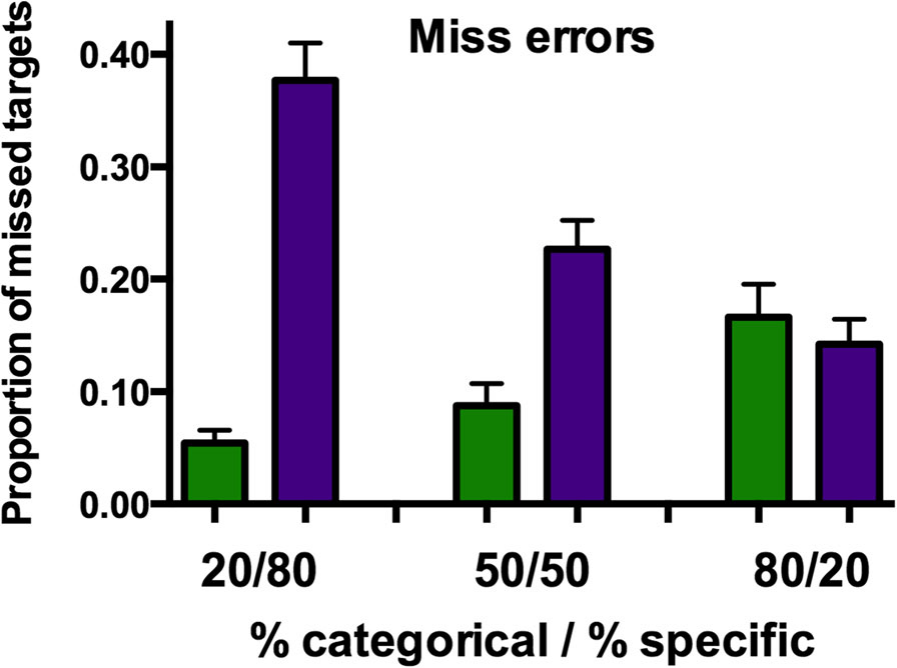
Miss error rates for the three conditions of Experiment 2. Notice that the most dramatic (seven times) differences between specific and categorical error rates occur when categorical targets are rare. Error bars represent +/- 1 s.e.m

The results are clear and in line with previous work on target prevalence in search (Baddeley & Colquhoun, 1969; Wolfe, 2012b; Wolfe & VanWert, 2010): as the probability of a target decreases, the chance that such targets will be missed increases. Rare categorical targets are missed more than common categorical targets and rare specific targets are missed more than common specific targets. The combination of this prevalence effect with the effects of mixing specific and categorical targets produces dramatic differences in error rates in the critical case of rare, categorical targets. Categorical targets are more than seven times more likely to be missed in the 20/80 condition, shown in Fig. 5.

## Experiment 3: Independent specific and categorical targets

In Experiments 1 and 2, the presence of specific targets is inversely related to the presence of categorical targets because a given trial has either one or the other type of target. It might have no target. It cannot have both. As a model of incidental findings, this is flawed since the presence of a broken rib does not rule out the possibility of pneumonia, for example. Accordingly, in Experiment 3, the presences of specific and categorical targets on a trial were independent of each other. This raises the possibility of multiple targets on each trial.

### Methods

As before, 12 observers (eight females, average age 30) memorized three specific and three categorical targets. With those targets committed to memory, they were tested on 1800 trials in six blocks of 300 trials with breaks in between. Visual set sizes were four, eight and 12 items. One target was present on 45% of trials. Two targets were present on 25% of trials. No targets were present on the remaining 30% of trials. Of the targets, 80% were specific and 20% were categorical. These constraints lead to the distribution of trials shown in Table 1.

**Table 1:** Distribution of trial types in Experiment 3

Constraints	
Absent trials	30%
One-target trials	45%
Two-target trials	25%
Categorical targets	20%
Specific targets	80%
Resulting trial distribution	
Absent	30%
One specific target	36%
One categorical target	9%
Two specific targets	16%
Two categorical targets	1%
One of each	8%
Total	100%

Observers responded by clicking on all of the targets that they found and then clicking on a separate box to indicate that they were finished with the trial.

### Results

Given the need to locate up to two targets and to make an explicit search termination response, trials were removed from analysis if the first or second RT was greater than 7000 msec or if the total RT for the trial was less than 200 or greater than 20,000 msec in length. This removed 0.5% of trials.

**Fig. 6:**
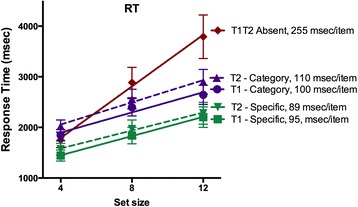
RT as a function of visual set size in Experiment 3. Note that search for the second (T2) categorical or specific target is essentially the same as search for the first (T1) item. Error bars represent +/- 1 s.e.m

The pattern of RTs is shown in Fig. 6.

It shows the same pattern of findings as the earlier experiments. It is somewhat harder to find categorical targets than to find specific targets. Target 2 (T2) RTs are measured from the time of the T1 response. Obviously, they are very similar to the T1 responses with, perhaps, a small increase in overall RT. Absent trials, as is typical in search experiments, are slower and increase more steeply with visual set size.

**Fig. 7:**
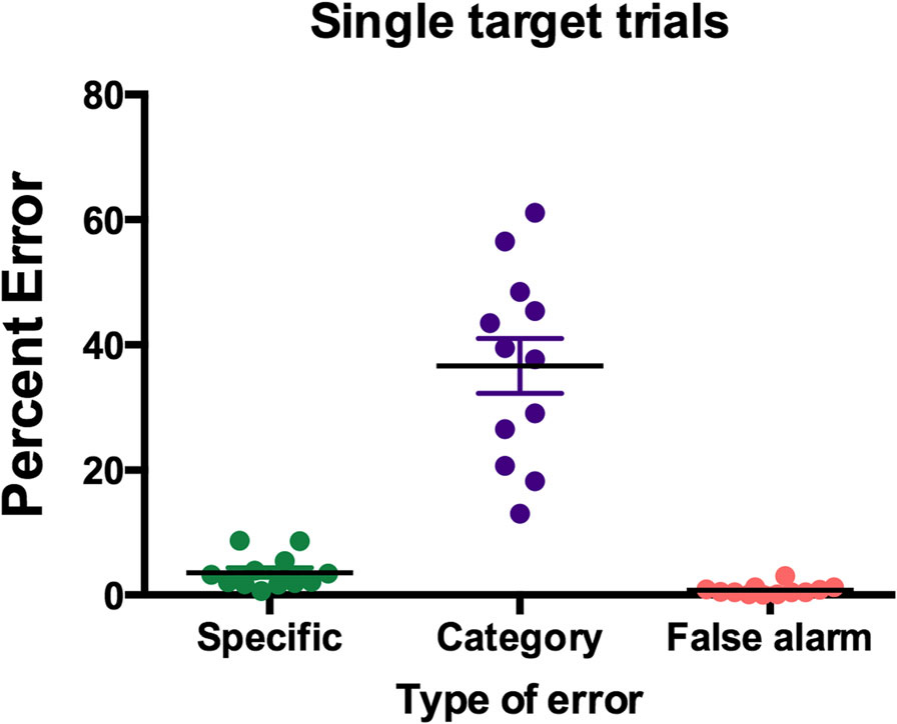
Errors for trials with a single specific or categorical target. Each dot represents the error rate for one observer

As in Experiments 1 and 2, the important data in Experiment 3 are the error rates. Figure 7 shows error rates for trials with either one or no target.

**Fig. 8:**
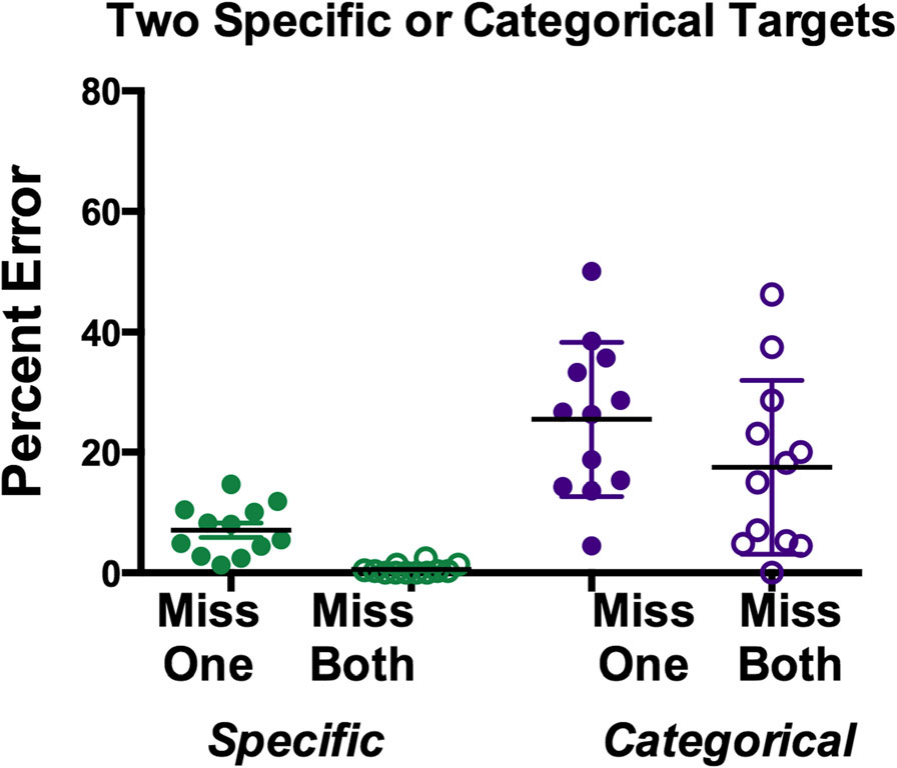
Miss errors for trials with two specific or categorical targets. Each dot represents the error rate for one observer. Open symbols represent trials where observers missed both instances of the target

Obviously, replicating Experiments 1 and 2, categorical targets are missed at a much higher rate (36.6%) than specific targets (3.6%), (t(11) = 9.1, *p* < 0.0001). Figure 8 shows the results when two categorical or two specific targets were present. Here we show the chance of missing one and the chance of missing both.

As above, many more categorical targets are missed (Miss one: t(11) = 9.4, *p* < 0.0001; Miss both: t(11) = 7.3, *p* < 0.0001). We can use the results in Fig. 7 to predict those in Fig. 8. Specifically, if we know the probability of finding one target, the probability of finding two, independent instances should be P(find 1) * P(find 1). For the specific targets, this works as expected. The average of the square of the chance of finding one target is 93.0% and the probability of finding two specific targets when two are present is 92.4%. The difference is not significant (t(11) = 0.9, *p* = 0.39). Interestingly, this is not true for categorical targets. The average of the square of the chance of finding one categorical target is 42.3%, but the probability of actually finding two categorical targets when two are present is 57%, (t(11) = 5.0, *p* = 0.0004). This may be an example of a reverse satisfaction of search effect (Berbaum et al., 1990; Berbaum et al., 2015). Normally, finding one target makes it less likely that a second will be found. Here, finding one, relatively rare categorical item may be reminding the observer that it might be worth look for a second.

**Fig. 9:**
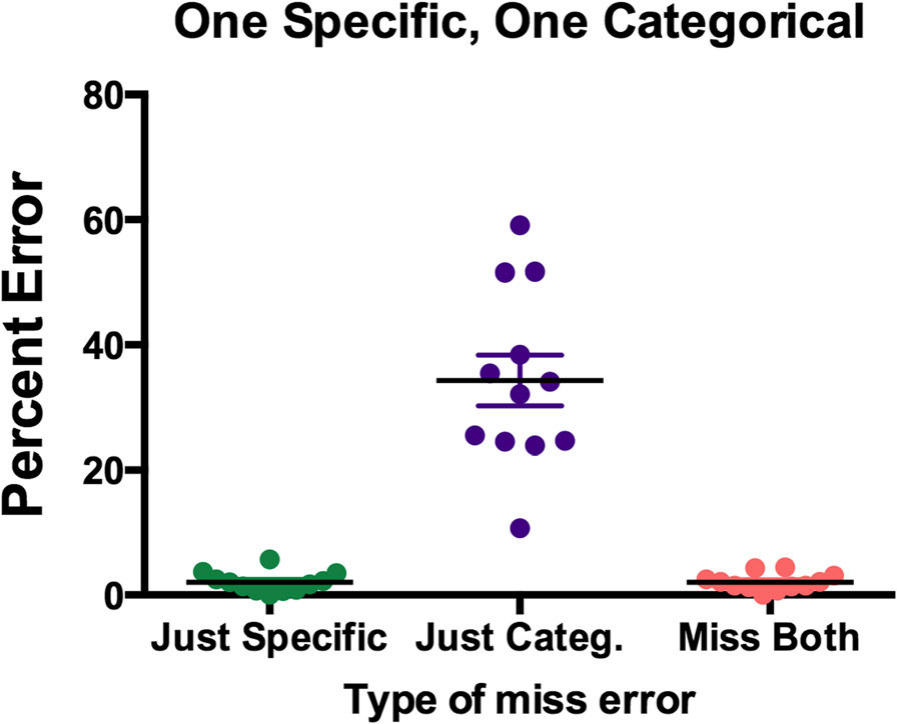
Miss errors for trials with one specific and one categorical target. Each dot represents the error rate for one observer

The most interesting case in Experiment 3 is the case where there are both categorical and specific targets in the same display. Figure 9 shows the pattern of errors in these trials.

Observers missed both targets 2.1% of the time. They missed only the specific target on another 2.1% of trials and missed only the categorical target on 34.8%. The difference is highly significant (t(11) = 8.5, *p* = 0.0001). On the 61% of trials where both items were found, the specific item was found first on 2/3rds of the cases (t(11) = 5.5, *p* = 0.0002). The probability of finding both items can be predicted from the probabilities of finding the single items: P(find 1 specific target) = 96.4%, P(categorical) = 63.4%; 96.4%*63.4% = 61.4%, actual P(find both) = 61.6%; t(11) = 0.8, *p* = 0.47). Thus, unlike the case of two categorical targets, finding a specific target does not appear to remind observers to look for a categorical target. The chance of finding both is equal to the product of finding each alone.

### Discussion

Overall, in Experiment 3, observers missed 4% of the specific targets and 35% of the categorical targets (t(11) = 8.9, *p* < 0.0001). Thus, Experiment 3 replicates the basic finding of the preceding experiments in this series. Categorical targets are missed at a much higher rate than specific targets. Indeed, the results are not dramatically different from those of Experiment 1 or 2, suggesting that the dependence or independence of categorical and specific targets is not a major driving force in these results. Experiment 3 does not find strong evidence for satisfaction of search (or SSM) effects in which the discovery of a specific target would have made discovery of a categorical target less likely. On trials where both specific and categorical targets are present, the rate at which categorical targets are missed is predicted by the rate at which those same targets would have been missed in the absence of a specific target. The one interaction between targets in this experiment seems to be a facilitation (effect) when both targets are categorical. The second categorical target is not missed quite as often as would be predicted.

## General discussion

The mixed hybrid search paradigm, introduced in this paper, appears to have promise as a ‘model system’ in which to study the problem of missed incidental findings in medicine. We can produce large and reliable rates of false negative errors in the search for targets that are known to the observer and searched for by the observer. This is an advance over, for example, our finding that radiologists miss a gorilla inserted into the lung (Drew et al., 2013). While it is interesting that most radiologists missed a gorilla, it must be conceded that they really were not looking for gorillas. In the present experiments, observers missed a third of items like “masks”, “signs”, or “furniture”, even though they knew perfectly well that they were looking for members of those categories. The errors in our experiments are not examples of what would typically be called “inattentional blindness” (Mack & Rock, 1998; Ward & Scholl, 2015). These are simply search errors; though these results and the analogy with incidental finding errors make it clear that simple search errors and inattentional blindness may be more closely related than we tend to think.

Is mixed hybrid search actually a good model of incidental finding errors? An answer to that question is beyond the scope of this paper, but the results of these experiments do provide a roadmap for future investigation into their relationship to the clinical situation we are trying to model. First, we did not find evidence of a satisfaction of search (or SSM) effect on trials where there was one specific and one categorical target. If mixed hybrid search is a model of incidental findings, then that should be true in the clinical setting as well. That is, the probability of reporting an incidental finding should be similar on cases that are positive or negative for the primary reason for the exam. This could be assessed though a review of existing cases.

Second, recall that observers did better than expected when there were two categorical targets. This suggests that the discovery of the first categorical target ‘primed’ the detection of the second (Kristjansson, Saevarsson, & Driver, 2013; Maljkovic & Nakayama, 1994). Perhaps we can reduce incidental finding errors by reminding observers to look for those loosely defined targets. Some priming effects can be quite long lasting (Kruijne & Meeter, 2016), so it is possible that showing observers the equivalent of these categorical targets at the start of a reading session might cut down on errors. This could be tested using our model system and then, if successful, tried in a clinical setting. This points to the basic motivation for this work. If mixed hybrid search is a good model for incidental finding errors, then interventions that bring down that >30% error rate in the lab might bring down the error rate in the clinic as well. Then, not only will we know more about the fundamental processes of visual search, we will be able to use that knowledge to improve medical care. One final note about reducing the incidental finding error rate: Ideally, we would want to reduce those errors without increasing the rates of false positive errors. We tend to focus on miss errors and, to be sure, missing a clinically significant finding is important. However, false positive errors carry their own costs. Moreover, in cases where the pathology is rare (e.g., cancer screening), a change in the false positive rate will affect many more people than a change of the same percentage in the miss error rate. Even if a single miss error is more consequential than a single false positive, a mass of false positives could, in principle, outweigh the benefits of fewer misses. In signal detection terms, our first goal should be to improve d’. If we are merely shifting criterion, we need to weigh the costs of one type of error against another.

## Conclusions

To briefly summarize, this paper introduces the “mixed hybrid search” paradigm, in which observers look through visual displays for any instance of several specific targets (“this boot”, “this screwdriver”, etc.) and several categorical targets (“any vegetable”, “any toy”, etc.). The key finding is that observers miss many more of the categorical targets, especially when the categorical targets are relatively rare. We propose this as a ‘model system’ for the problem of incidental findings in radiology. If we can manipulate the errors in the lab, we may be able to do the same in the clinic.

## References

[CR1] Adamo, S. H., Cain, M. S., & Mitroff, S. R. (2013). Self-induced attentional blink: a cause of errors in multiple-target search. *Psychological Science, 12.* doi:10.1177/0956797613497970 10.1177/0956797613497970 24142814

[CR2] Baddeley AD, Colquhoun WP (1969). Signal probability and vigilance: a reappraisal of the ‘signal-rate’ effect. British Journal of Psychology.

[CR3] Beigelman-Aubry C, Hill C, Grenier PA (2007). Management of an incidentally discovered pulmonary nodule. European Radiology.

[CR4] Berbaum KS, Brandser EA, Franken EA, Dorfman DD, Caldwell RT, Krupinski EA (2001). Gaze dwell times on acute trauma injuries missed because of satisfaction of search. Academic Radiology.

[CR5] Berbaum KS, Franken EA, Caldwell RT, Shartz K, Krupinski EA, Samei E (2010). Satisfaction of search in traditional radiographic imaging. The Handbook of Medical Image Perception and Techniques.

[CR6] Berbaum KS, Franken EA, Dorfman DD, Rooholamini SA, Coffman CE, Cornell SH (1991). Time course of satisfaction of search. Investigative Radiology.

[CR7] Berbaum KS, Franken EA, Dorfman DD, Rooholamini SA, Kathol MH, Barloon TJ (1990). Satisfaction of search in diagnostic radiology. Investigative Radiology.

[CR8] Berbaum KS, Krupinski EA, Schartz KM, Caldwell RT, Madsen MT, Hur S (2015). Satisfaction of search in chest radiography 2015. Academic Radiology.

[CR9] Berlin L (2016). Re:“Rethinking normal: Benefits and risks of not reporting harmless incidental findings. Journal of the American College of Radiology.

[CR10] Boettcher S, Wolfe JM (2015). Searching for the right word: Hybrid visual and memory search for words. Attention, Perception & Psychophysics.

[CR11] Bos D, Poels MMF, Adams HHH, Akoudad S, Cremers LGM, Zonneveld HI (2016). Prevalence, clinical management, and natural course of incidental findings on brain MR images: the population-based Rotterdam Scan Study. Radiology.

[CR12] Cain MS, Adamo SH, Mitroff SR (2013). A taxonomy of errors in multiple-target visual search. Visual Cognition.

[CR13] Castel AD, Vendetti M, Holyoak K (2012). Fire drill: Inattentional blindness and amnesia for the location of fire extinguishers. Attention, Perception & Psychophysics.

[CR14] Chabris CF, Simons DJ (2011). You do not talk about Fight Club if you do not notice Fight Club: Inattentional blindness for a simulated real-world assault. i-Perception.

[CR15] Chun MM, Wolfe JM (1996). Just say no: How are visual searches terminated when there is no target present?. Cognitive Psychology.

[CR16] Cohen MA, Cavanagh P, Chun MM, Nakayama K (2012). The attentional requirements of consciousness. Trends in Cognitive Sciences.

[CR17] Cunningham CA, Wolfe JM (2014). The role of object categories in hybrid visual and memory search. Journal of Experimental Psychology: General.

[CR18] Dalton P, Fraenkel N (2012). Gorillas we have missed: Sustained inattentional deafness for dynamic events. Cognition.

[CR19] Drew T, Vo ML-H, Wolfe JM (2013). The invisible gorilla strikes again: sustained inattentional blindness in expert observers. Psychological Science.

[CR20] Hogg RV, Craig AT (1995). Introduction to mathematical statistics.

[CR21] Kristjansson Å, Saevarsson S, Driver J (2013). The boundary conditions of priming of visual search: From passive viewing through task-relevant working memory load. Psychonomic Bulletin & Review.

[CR22] Kruijne W, Meeter M (2016). Long-term priming of visual search prevails against the passage of time and counteracting instructions. Journal of Experimental Psychology: Learning, Memory, and Cognition.

[CR23] Lumbreras B, Donat L, Hernández-Aguado I (2010). Incidental findings in imaging diagnostic tests: a systematic review. The British Journal of Radiology.

[CR24] Mack A, Rock I (1998). Inattentional Blindness.

[CR25] Maljkovic V, Nakayama K (1994). Priming of popout: I. Role of features. Memory & Cognition.

[CR26] Maxfield JT, Zelinsky GJ (2012). Searching through the hierarchy: How level of target categorization affects visual search. Visual Cognition.

[CR27] Moran, R., Zehetleitner, M., Liesefeld, H., Müller, H., & Usher, M. (2015). Serial vs. parallel models of attention in visual search: accounting for benchmark RT-distributions. *Psychonomic Bulletin & Review*, 1-16. doi: 10.3758/s13423-015-0978-1. 10.3758/s13423-015-0978-1 26635097

[CR28] Nako R, Wu R, Smith TJ, Eimer M (2014). Item and category-based attentional control during search for real-world objects: Can you find the pants among the pans?. Journal of Experimental Psychology: Human Perception and Performance.

[CR29] Nosofsky RM, Cao R, Cox GE, Shiffrin RM (2014). Familiarity and categorization processes in memory search. Cognitive Psychology.

[CR30] Nosofsky RM, Cox GE, Cao R, Shiffrin RM (2013). Memory search with short and long lists viewed from the perspective of an exemplar-familiarity model.

[CR31] Pandharipande, P. V., Herts, B. R., Gore, R. M., Mayo-Smith, W. W., Harvey, H. B., Megibow, A. J., et al. (2016a). Authors’ Reply. *Journal of the American College of Radiology, 13*, 1025-1027. doi: 10.1016/j.jacr.2016.06.045. 27593092

[CR32] Pandharipande, P. V., Herts, B. R., Gore, R. M., Mayo-Smith, W. W., Harvey, H. B., Megibow, A. J., et al. (2016b). Rethinking normal: Benefits and risks of not reporting harmless incidental findings. *Journal of the American College of Radiology*, S1546-1440(1516)30108-30109. doi: 10.1016/j.jacr.2016.03.017. 27162042

[CR33] Schneider W, Shiffrin RM (1977). Controlled and automatic human information processing: I. Detection, search, and attention. Psychological Review.

[CR34] Schwarz, W., & Miller, J. O. (2016). GSDT: An integrative model of visual search. *Journal of Experimental Psychology Human Perception and Performance, 42*, 1654-1616-1675. Advance online publication. 10.1037/xhp0000247. 27336631

[CR35] Simons DJ, Chabris CF (1999). Gorillas in our midst: sustained inattentional blindness for dynamic events. Perception.

[CR36] Tuddenham WJ (1962). Visual search, image organization, and reader error in roentgen diagnosis. Studies of the psycho-physiology of roentgen image perception. Radiology.

[CR37] Ward EJ, Scholl BJ (2015). Inattentional blindness reflects limitations on perception, not memory: Evidence from repeated failures of awareness. Psychonomic Bulletin and Review.

[CR38] Wolfe, J. M. (2012a). Saved by a log: How do humans perform hybrid visual and memory search? *Psychological Science, 23*, 698-703. doi:10.1177/0956797612443968 10.1177/0956797612443968 PMC3966104 22623508

[CR39] Wolfe, J. M. (2012b). When do I quit? The search termination problem in visual search. *Nebraska Symposium on Motivation, 59*, 183-208 10.1007/978-1-4614-4794-8_8 PMC3979292 23437634

[CR40] Wolfe JM, Boettcher SEP, Josephs EL, Cunningham CA, Drew T (2015). You look familiar, but I don’t care: Lure rejection in hybrid visual and memory search is not based on familiarity. Journal of Experimental Psychology: Human Perception and Performance.

[CR41] Wolfe JM, VanWert MJ (2010). Varying target prevalence reveals two, dissociable decision criteria in visual search. Current Biology.

[CR42] Yang H, Zelinsky GJ (2009). Visual search is guided to categorically defined targets. Vision Research.

